# A DATA-BASED PROPOSITION FOR THE CREATION OF NEW ICD-10 DIAGNOSIS CODES FOR POST-ACUTE SEPSIS SURVIVORS

**DOI:** 10.1093/geroni/igac059.1798

**Published:** 2022-12-20

**Authors:** Sungho Oh, Melissa O’Connor, Michael Stawnychy, Yolanda Barrón, Patrik Garren, Elaine Sang, Kathryn Bowles

**Affiliations:** University of Pennsylvania, Philadelphia, Pennsylvania, United States; Villanova University, M. Louise Fitzpatrick College of Nursing, Villanova, Pennsylvania, United States; University of Pennsylvania School of Nursing, Philadelphia, Pennsylvania, United States; Visiting Nurse Service of New York, New York, New York, United States; University of Pennsylvania, Philadelphia, Pennsylvania, United States; University of Pennsylvania, Philadelphia, Pennsylvania, United States; University of Pennsylvania School of Nursing, Philadelphia, Pennsylvania, United States

## Abstract

Sepsis survivors have the second highest readmission rate among Medicare beneficiaries, next to heart failure. I-TRANSFER is an implementation science study to improve transitions and reduce readmissions among sepsis survivors transitioning from acute to home health care (HHC). A total of 63 semi-structured interviews were conducted with stakeholders among 12 hospitals and 5 affiliated home health agencies (HHAs). The purpose of this secondary analysis was to: 1) examine how the sepsis diagnosis is reflected in HHC documentation, 2) identify barriers in sepsis information transfer to HHC, 3) recommend a documentation strategy to enhance information transfer and HHC documentation. We analyzed the diagnosis coding within a national OASIS dataset and eight I-TRANSFER interviews with hospitalists, documentation specialists, and HHC coders. Findings include: a) sepsis diagnoses were nearly invisible in HHC records being documented for only 10% of 165,000 sepsis survivors transitioned to HHC, b) sepsis information in referral documentation can be unclear to HHC coders, c) the lack of sepsis diagnosis documentation might make HHC clinicians unaware of the patient’s risk for readmission, d) HHC coders recommended improved language in the acute care discharge summary to link the need for HHC to sepsis, thereby supporting the use of ICD-10 sepsis ‘A’ codes in HHC. The use of terms, such as “History of Sepsis” or “Sepsis Resolved,” leads to HHC using non-specific codes, thus is not recommended. We highlight the need for new ICD-10 codes for “Sepsis Aftercare” and “Post-Sepsis Syndrome” to clearly communicate and document the needs of sepsis survivors.

